# Supplementary material for: Transposable elements as tissue-specific enhancers in cancers of endodermal lineage
Source: Nat Commun. 2023 Sep 1;14:5313. doi: 10.1038/s41467-023-41081-4 (PMC10474299; doi:10.1038/s41467-023-41081-4)
Supplement: Supplementary file 1 — Supplementary Information [file 41467_2023_41081_MOESM1_ESM.pdf]

Supplementary Figures 1-13 for:

Transposable elements as tissue-specific enhancers in endodermal-lineage cancers

Konsta Karttunen[1*], Divyesh Patel[1,2*], Jihan Xia[1,2], Liangru Fei[1], Kimmo Palin[1,2], Lauri Aaltonen[1,2] and Biswajyoti Sahu[1,2,3,4#]

[1]Applied Tumor Genomics Program, Research Programs Unit, Faculty of Medicine, University of Helsinki, Helsinki, Finland

[2]iCAN Digital Precision Cancer Medicine Flagship, University of Helsinki, Helsinki, Finland

[3]Medicum, Faculty of Medicine, University of Helsinki, Helsinki, Finland

[4]Centre for Molecular Medicine Norway, University of Oslo, Oslo, Norway

* equal contribution

# Corresponding author: Biswajyoti Sahu (biswajyoti.sahu@helsinki.fi)

Supplementary Figure 1

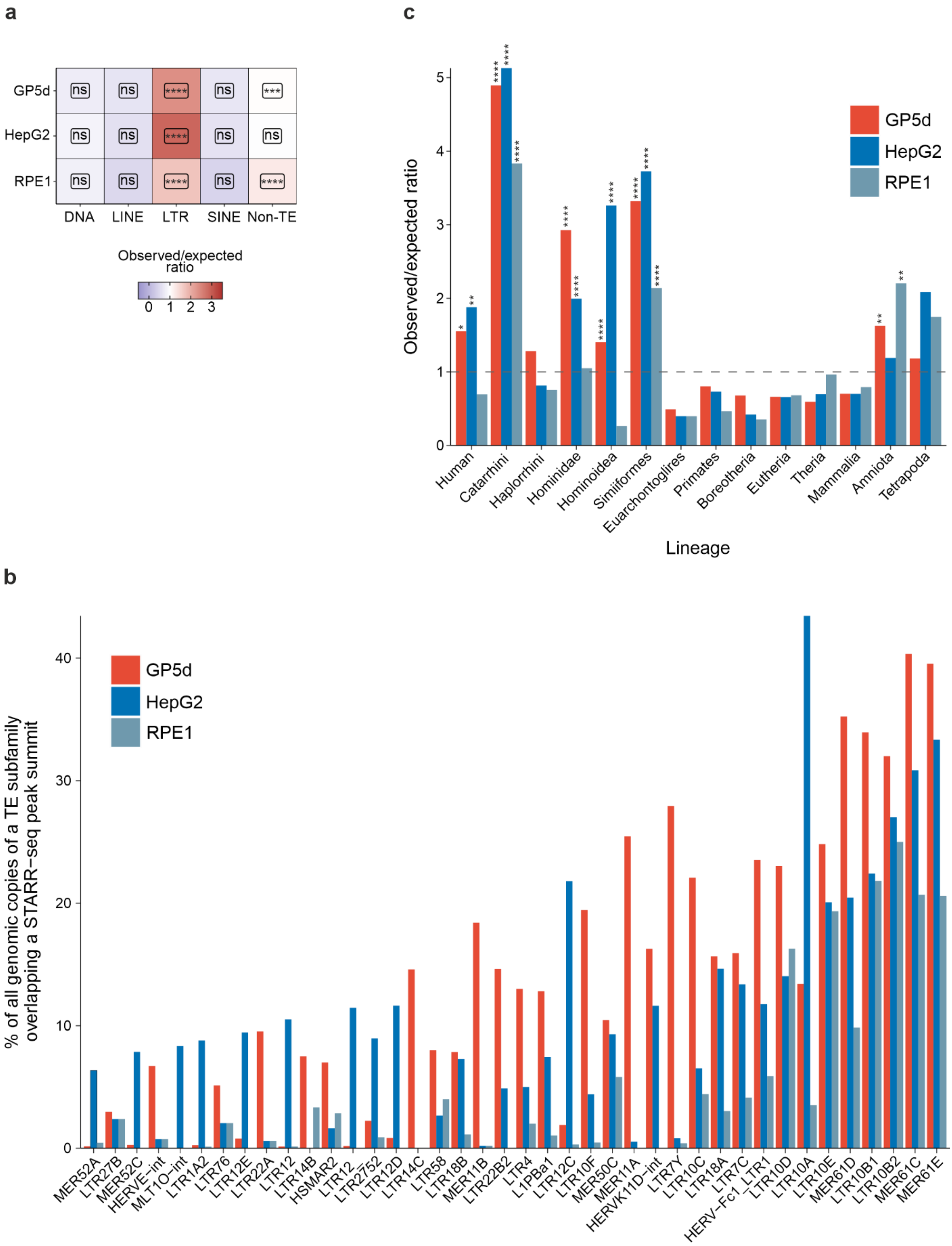

Supplementary Figure 1 | Overlap of TE subfamilies with active enhancers in GP5d, HepG2 and RPE1 cells identified by STARR-seq. a, Enrichment of TE classes overlapping a STARR-seq peak summit in GP5d, HepG2 and RPE1 cells. BH-adjusted one-sided binomial test FDR is shown for each class (Significance symbols: **** = p < 0.0001, *** = p < 0.001, ** = p < 0.01, * = p < 0.05, ns = non-significant, p > 0.05). GP5d LTR p < 2.2e-16 and non-TE p

= 4.331607e-04, HepG2 LTR p < 2.2e-16, RPE1 LTR p < 2.2e-16 and non-TE < 2.2e-16. n = 15391, 11956, and 6476 for tested GP5d, HepG2 and RPE1 STARR-seq peak summits, respectively. . **b,** Percentages of all genomic copies of each TE subfamily overlapping a STARR-seq peak summit. Top 40 subfamilies with the highest mean percentages across the cell lines are shown. Number of STARR-seq peak summits are as in panel **a.**. **c,** Enrichment of TEs overlapping STARR-seq peak summits classified by lineage of origin for the TE subfamilies. Lineage data from Supplementary ref. [1]. Significance symbols as in panel **a** are shown for BH-adjusted one-sided binomial test FDR. Human GP5d p = 1.271258e-02, HepG2 p = 8.866981e-04, Catarrhini GP5d p < 2.2e-16, HepG2 p < 2.2e-16, RPE1 p < 2.2e-16, Hominidae GP5d p < 2.2e-16, HepG2 p = 2.706703e-08, Hominoidea GP5d p = 1.274746e-12, HepG2 p < 2.2e-16, Simiiformes GP5d p < 2.2e-16, HepG2 p < 2.2e-16 , RPE1 p < 2.2e-16, Amniota GP5d p = 6.966019e-03, RPE1 p = 3.251458e-03. Number of tested STARR-seq peak summits are as in panel **a.** Source data are provided as a Source Data file.

Supplementary Figure 2

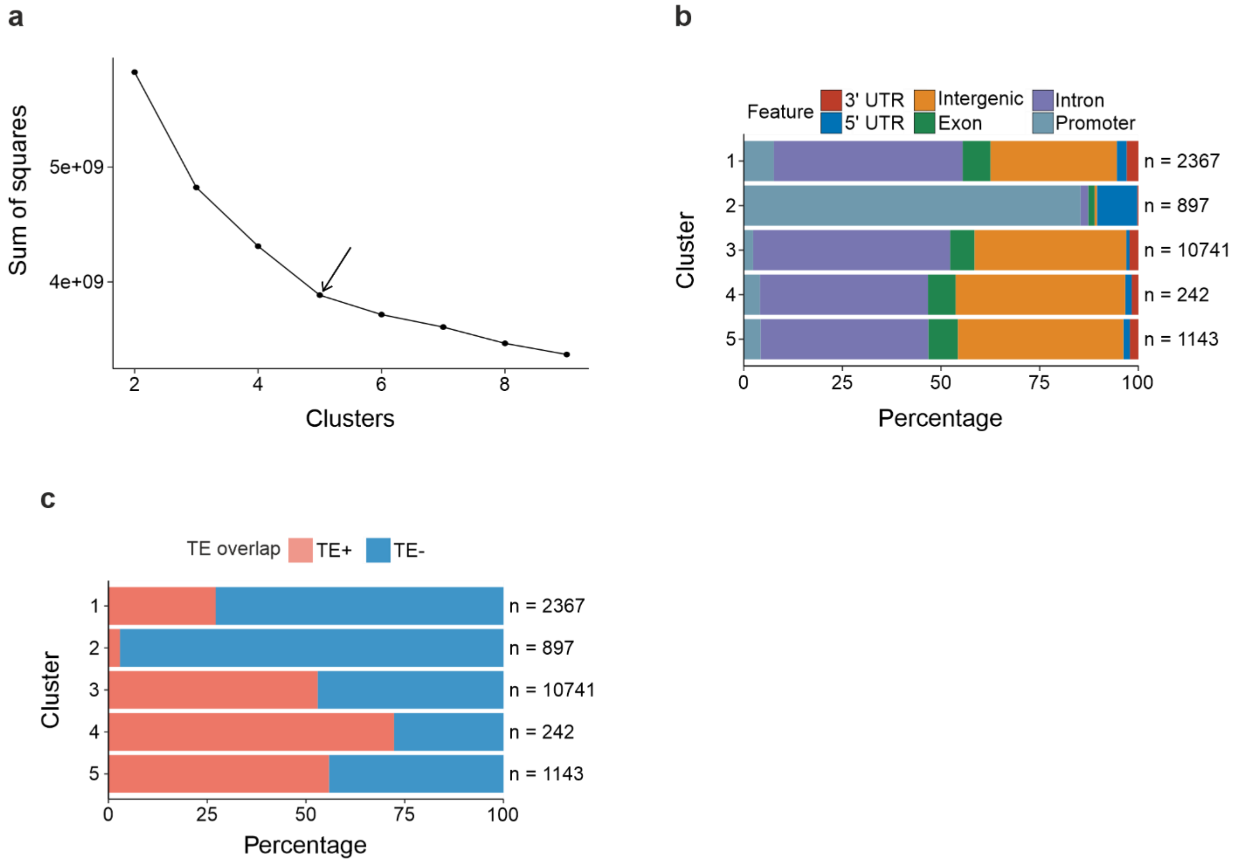

Supplementary Figure 2 | GP5d STARR-seq peak clustering details. a, Elbow plot of k-means clustering for GP5d STARR-seq peaks showing the elbow point at five clusters (indicated with the arrow). Thus, five clusters was used in the analysis shown in **Fig. 2a**. **b,** Distribution of peaks within the five clusters from **Fig. 2a** in different genomic regions. n = 2367, 897, 10741, 242, and 1143 for clusters 1-5, respectively. UTR = untranslated region. **c,** Percentages of STARR-seq peaks within the five clusters from **Fig. 2a** overlapping TEs. Number of peaks in clusters are as in panel **b.** Source data are provided as a Source Data file.

Supplementary Figure 3

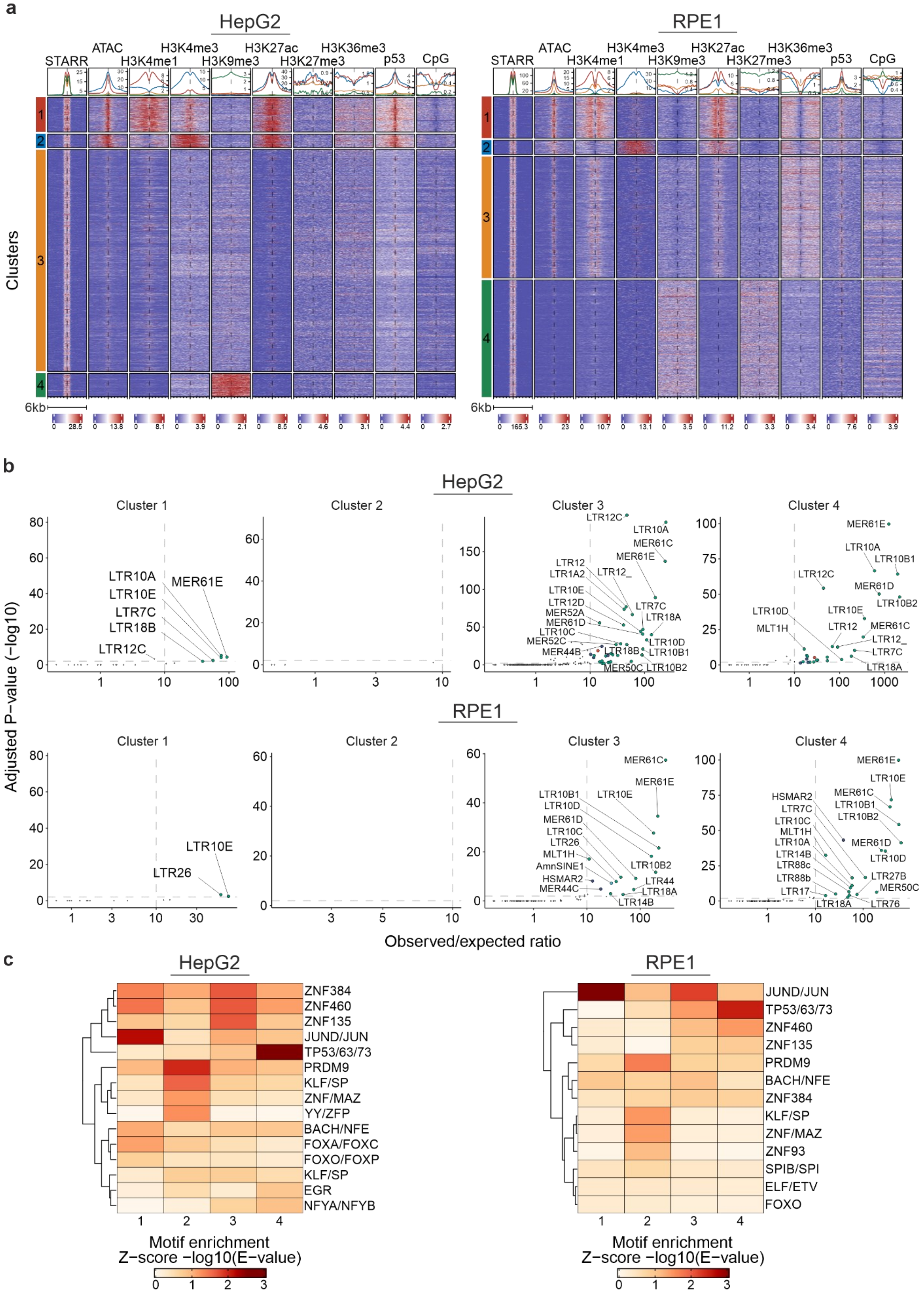

Supplementary Figure 3 | Clustering of STARR-seq and epigenetic data and TE enrichment analysis in HepG2 and RPE1 cells. a, Clustering of STARR-seq and epigenetic data in HepG2 and RPE1 cells as in **Fig. 2a**. Four clusters resulted in an optimal number of clusters for both cell lines. **b,** Enrichment of TEs in each cluster for HepG2 and RPE1 as in **Fig. 2c**. **c,** TF motif enrichment in each cluster for HepG2 and RPE1 as in **Fig. 2b**. After performing the motif enrichment analysis for individual motifs, similar motifs were combined into motif clusters according to Supplementary ref. [2]. The representative TF family and motif are shown for each clustered motif. Source data are provided as a Source Data file.

Supplementary Figure 4

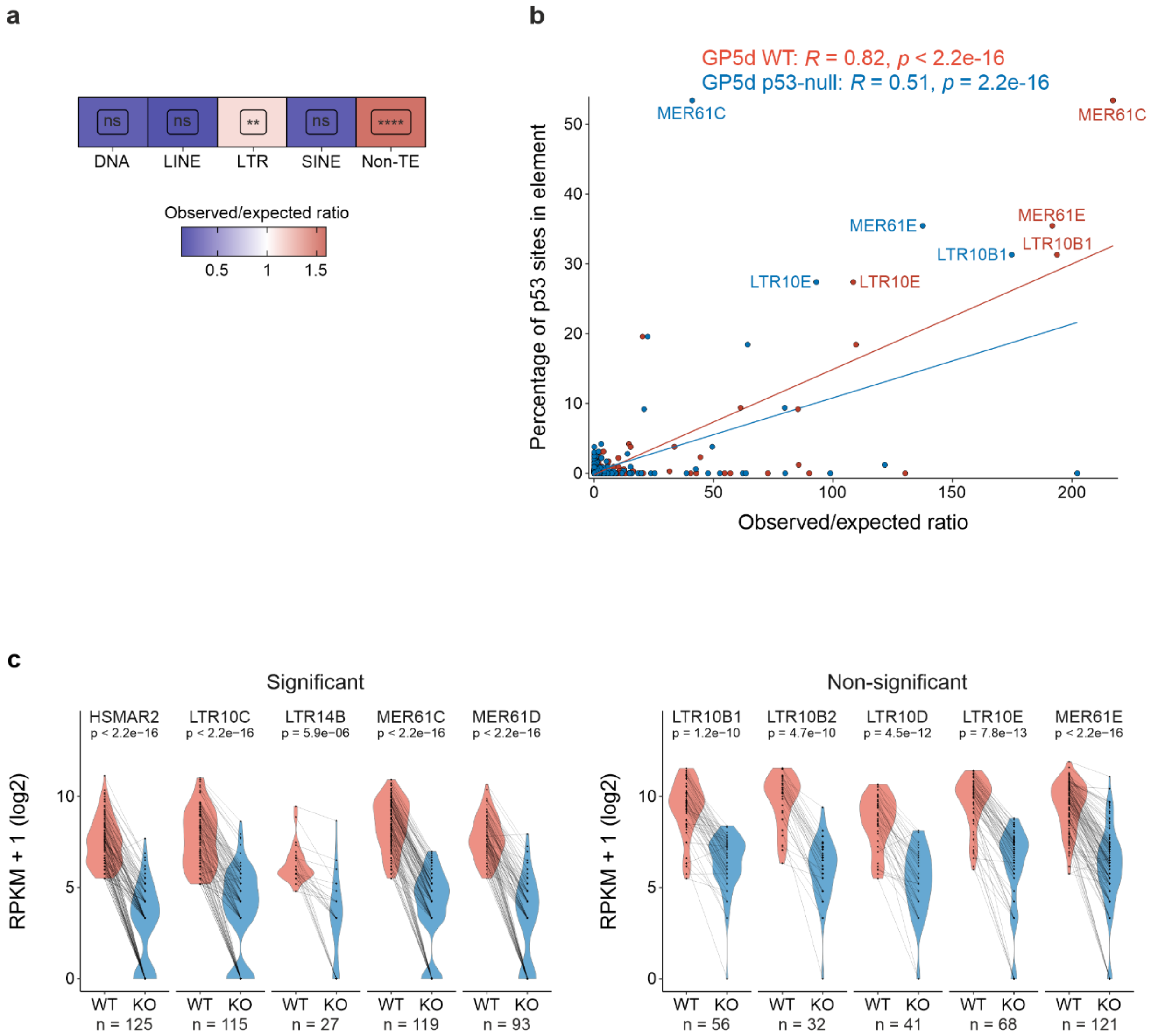

Supplementary Figure 4 | Details of p53-specificity in wild type and p53-null GP5d. a, Enrichment of different TE classes within p53 peak summits overlapping TEs. BH-adjusted one-sided binomial test FDR is shown for each class (Significance symbols: **** = $p < 0.0001$, *** = $p < 0.001$, ** = $p < 0.01$, * = $p < 0.05$, ns = non-significant, $p > 0.05$). LTR p = 0.009413791 and non-TE p < 2.2e-16. n = 4297 for p53 ChIP-seq peak summits. **b,** Correlation of observed/expected ratio STARR-seq peak summit overlap and percentage of TE copies in a subfamily with a p53 response element in wild type and p53-null GP5d. p53 motif predictions were obtained from Supplementary ref. [3]. GP5d WT Pearson's R = 0.82, p < 2.2e-16, GP5d p53-null Pearson's R = 0.51, p < 2.2e-16. n = 15391 and 13349 for tested GP5d WT and GP5d p53-null STARR-seq peak summits, respectively. **c,** STARR-seq signal at p53-specific TEs. Log_2-transformed RPKM values were calculated at all STARR-seq peaks overlapping TEs in GP5d WT (WT) and p53-null (KO). Left panel shows TE subfamilies that were significantly more enriched in WT (main **Fig. 2d**), and right side shows representative subfamilies of p53-specific TEs that did not show a significant difference (selected on the basis of motif analysis in main **Fig. 3b**). . P-values were calculated with a two-sided, paired Wilcoxon test. HSMAR2 p < 2.2e-16, LTR10C p < 2.2e-16, LTR14B p = 5.9e-06, MER61C p < 2.2e-16, MER61D p < 2.2e-16. LTR10B1 p = 1.2e-10, LTR10B2 p = 4.7e-10, LTR10D p = 4.5e-12, LTR10E p = 7.8e-13, MER61E p < 2.2-16. n = 125, 115, 27, 119, and 93 for HSMAR2, LTR10C, LTR14B,

MER61C, and MER61D, respectively. n = 56, 32, 41, 68, and 121 for LTR10B1, LTR10B2, LTR10D, LTR10E, and MER61E, respectively Source data are provided as a Source Data file.

Supplementary Figure 5

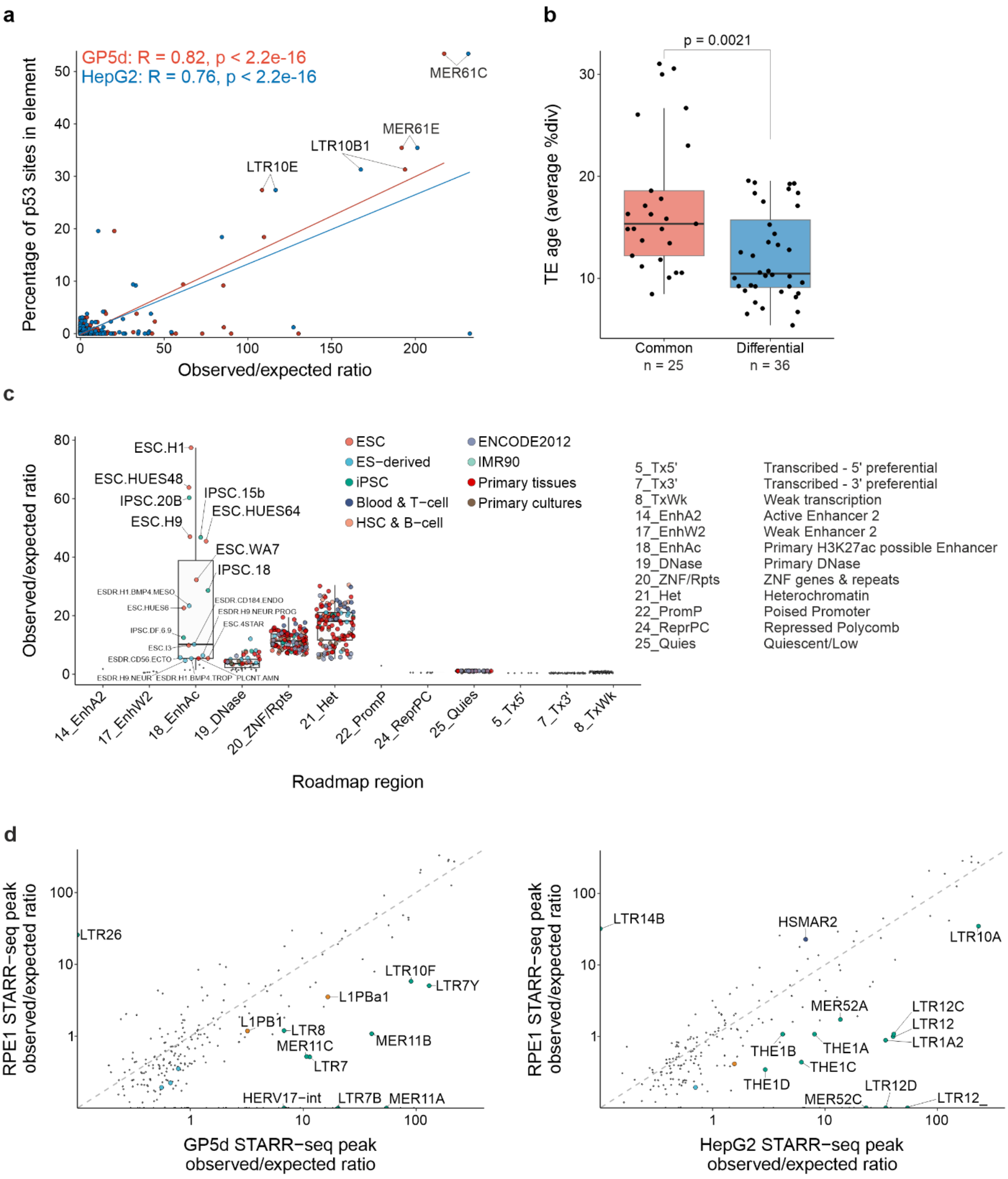

Supplementary Figure 5 | Differential enrichment of TE subfamilies in GP5d and HepG2 cells. a, Correlation of observed/expected ratio STARR-seq peak summit overlap and percentage of TE copies in a subfamily with a p53 response element in GP5d wild type and HepG2. p53 site predictions were obtained from Supplementary ref. [3]. GP5d Pearson's R = 0.82, p < 2.2e-16, HepG2 Pearson's R = 0.76, p < 2.2e-16. n = 15391 and 11956 for tested GP5d and HepG2 STARR-seq peak summits, respectively. **b,** The average age of commonly

vs. differentially enriched TE subfamilies between GP5d and HepG2 cells. Two-sided Wilcoxon test p = 0.0021. n = 25 and 36 common and differential subfamilies, respectively. The lower and upper hinges of the boxes represent the 25th to 75th percentiles, the midline is the median, and the whiskers extend from the hinges to the minimum and maximum values by 1.5 * interquartile range. **c,** Enrichment of chromatin states in 127 Roadmap tissues at GP5d STARR-seq peak summits overlapping MER11 elements in the 25-state chromatin model. Chromatin state for 127 Roadmap tissues were obtained from Supplementary ref. [4]. Chromatin states with a minimum of three overlapping summits in a cell type are shown. Plot elements are similar as in figure **b**. n = 398 MER11 elements overlapping STARR-seq peak summits tested for each tissue. **d,** Differential enrichment of TEs as in main **Fig. 3a**. Left panel is TE enrichment RPE1 Vs GP5d and right panel is RPE1 vs. HepG2. Source data are provided as a Source Data file.

Supplementary Figure 6

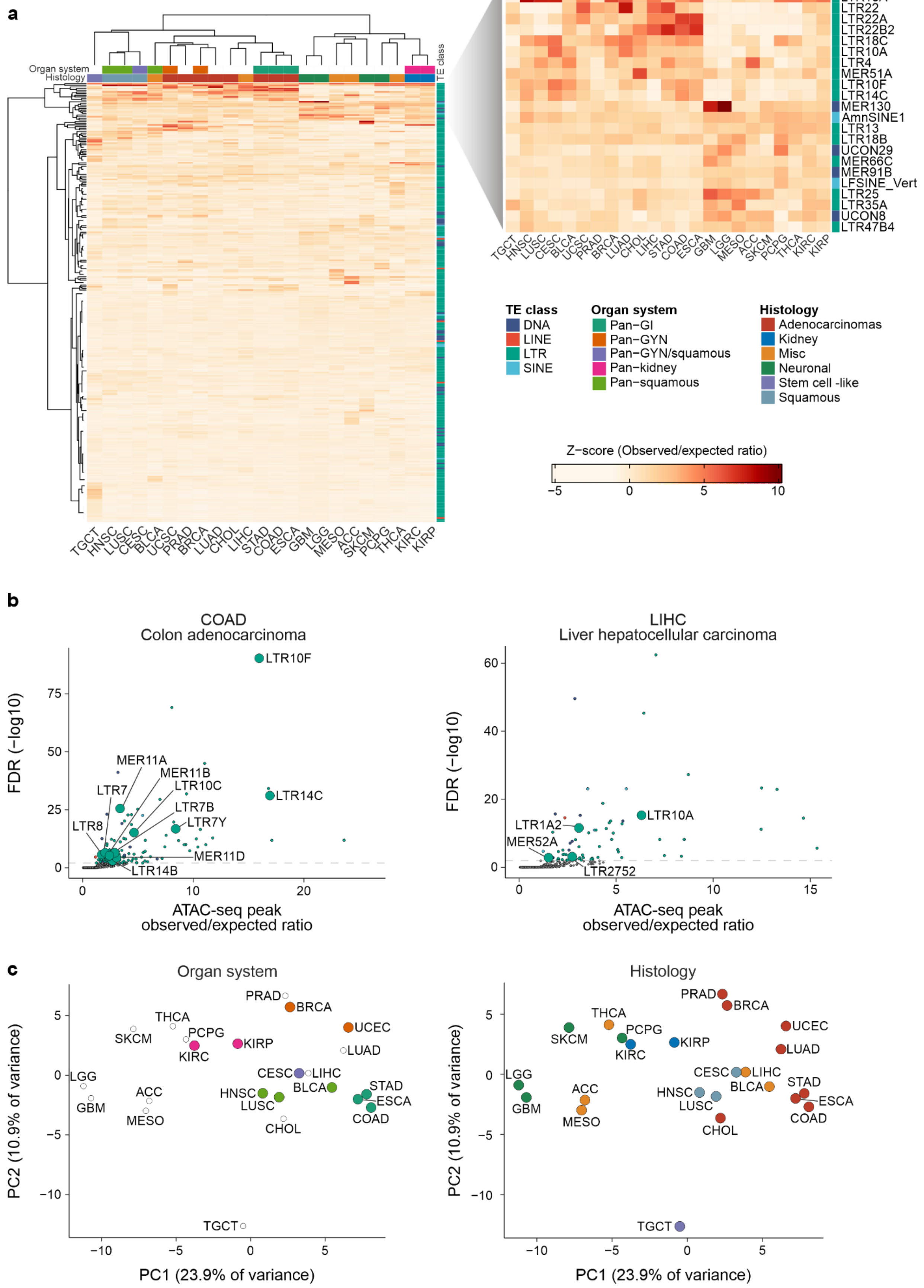

Supplementary Figure 6 | TE enrichment within open chromatin regions in human tumor samples. a, Enrichment of TE subfamilies was calculated within cancer type-specific ATAC-seq peak in 23 TCGA cancer types from datasets acquired from Supplementary ref. [5]. Top 23 most enriched TE subfamilies across cancer types are highlighted in the panel on the right. Rows and columns are clustered with hierarchical clustering with Euclidean distances and Ward linkage. The abbreviations used for the TCGA cancer types are as follows in alphabetical order: ACC: Adrenocortical carcinoma, BLCA: Bladder Urothelial Carcinoma, BRCA: Breast invasive carcinoma, CESC: Cervical squamous cell carcinoma and endocervical adenocarcinoma, CHOL: Cholangiocarcinoma, COAD: Colon adenocarcinoma, ESCA: Esophageal carcinoma, GBM: Glioblastoma multiforme, HNSC: Head and Neck squamous cell carcinoma, KIRC: Kidney renal clear cell carcinoma, KIRP: Kidney renal papillary cell carcinoma, LGG: Brain Lower Grade Glioma, LIHC: Liver hepatocellular carcinoma, LUAD: Lung adenocarcinoma, LUSC: Lung squamous cell carcinoma, MESO: Mesothelioma, PCPG: Pheochromocytoma and Paraganglioma, PRAD: Prostate adenocarcinoma, SKCM: Skin Cutaneous Melanoma, STAD: Stomach adenocarcinoma, TGCT: Testicular Germ Cell Tumors, THCA: Thyroid carcinoma, and UCEC: Uterine Corpus Endometrial Carcinoma**.** Source data are provided as a Source Data file. **b,** Scatterplots of TE subfamily enrichment within ATAC-seq peaks in TCGA colorectal and hepatocellular cancer types. Significantly enriched TE subfamilies (one-sided binomial test FDR < 0.01) that were also differentially enriched in respective colorectal (GP5d) and hepatocellular (HepG2) cell lines from **Fig. 3a** are highlighted and labelled in the plots. **c,** Principal component analysis of TE enrichment observed/expected ratios within ATAC-seq peaks in the 23 TCGA cancer types, with organ systems of origin labelled as in Supplementary ref. [6] in the left panel and histology labelled as in Supplementary ref. [7] in the right panel (color codes for different organ systems and histological types as in panel **a**). Source data are provided as a Source Data file.

Supplementary Figure 7

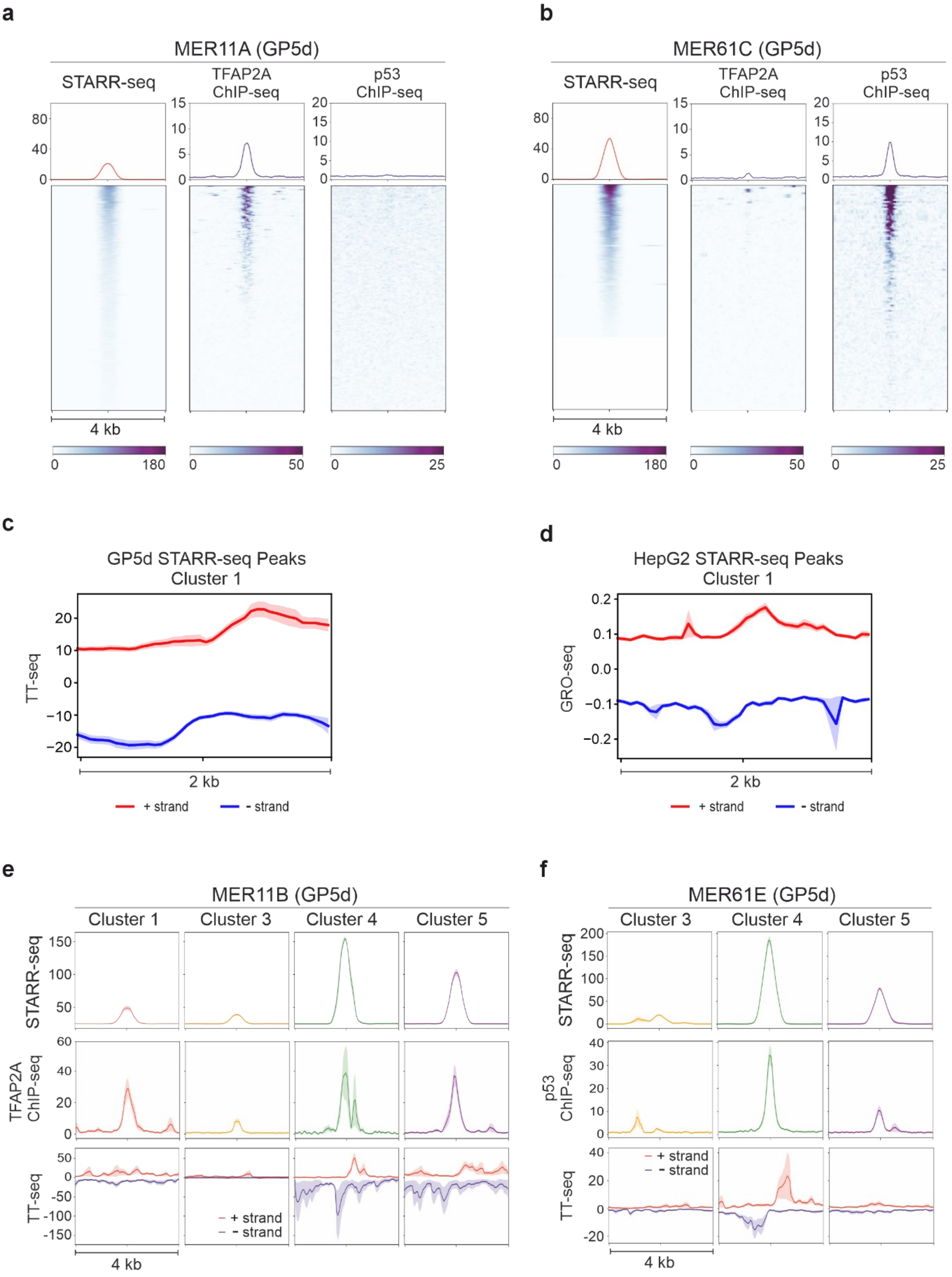

Supplementary Figure 7 | Episomal enhancer activity from STARR-seq correlates with nascent transcriptional activity from genomic MER11B and MER61C elements in GP5d. a, GP5d STARR-seq peaks overlapping with annotated MER11A repeats were extracted.

Heatmap shows signal of STARR-seq, TFAP2A and p53 ChIP-seq in flanks of MER11A containing STARR-seq peaks in GP5d cells. **b,** GP5d STARR-seq peaks overlapping with annotated MER61C repeats were extracted. Heatmap shows signals for STARR-seq, TFAP2A and p53 ChIP-seq in flanks of MER61C containing STARR-seq peaks in GP5d cells. **c and d,** TT-seq and GRO-seq signal plotted in a ±1kb region from the center of the cluster 1 STARR-seq peaks from GP5d from main **Fig. 2a** and HepG2 from **Supplementary Figure 3a**, respectively. Metaplots show the average signal with standard error. **e,** Metaplots comparing the STARR-seq, TFAP2A ChIP-seq and TT-seq signal at MER11B loci containing STARR-seq peaks representing the four clusters from main **Fig. 2a**. **f,** Metaplots compare the STARR-seq, p53 ChIP-seq and TT-seq signal at MER61E containing STARR-seq peaks representing the three clusters from **Fig. 2a**. All metaplots show the average signal with standard error.

Supplementary Figure 8

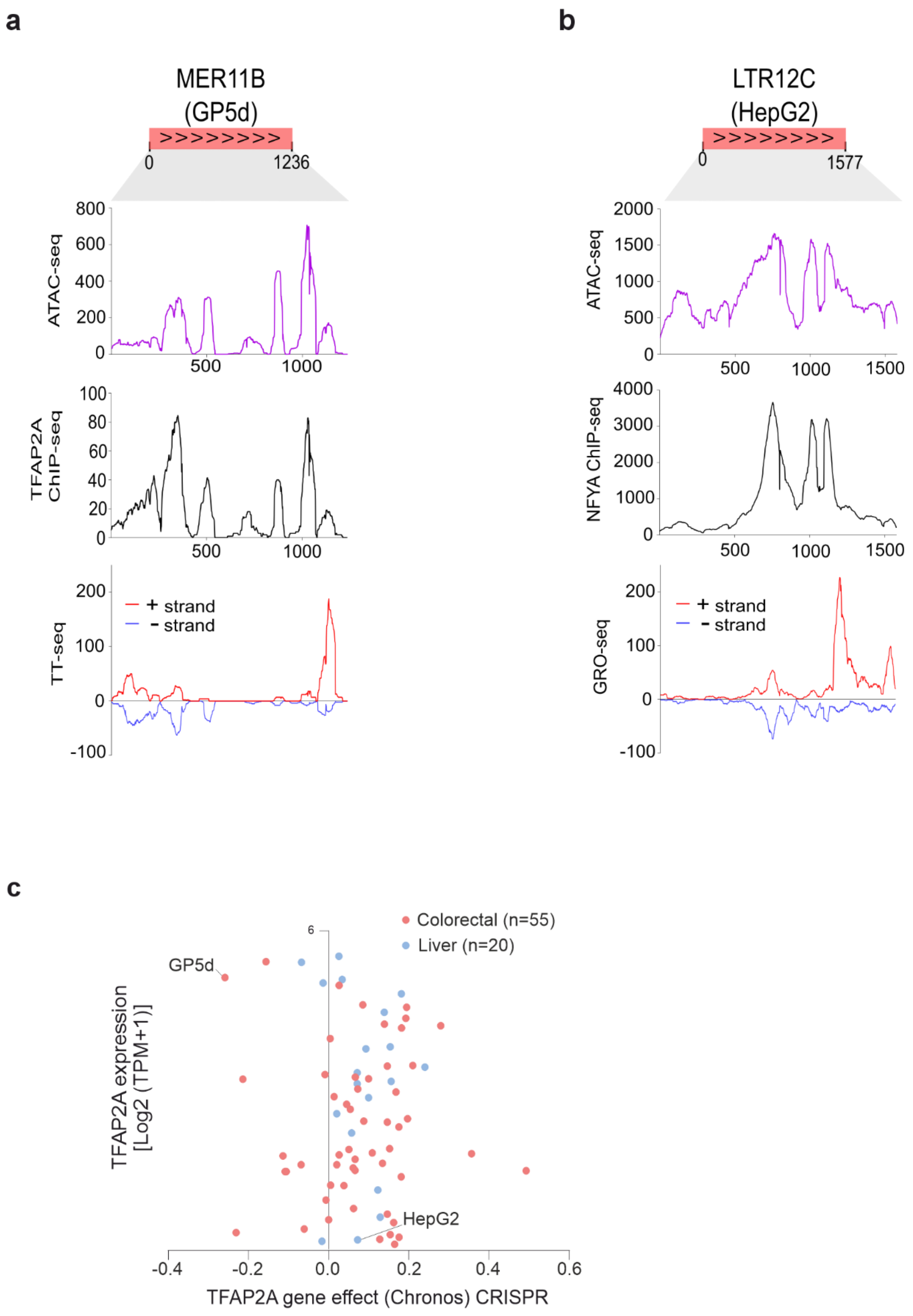

Supplementary Figure 8 | Nascent transcriptional activity correlates with TF binding and chromatin accessibility at MER11B and LTR12C elements. a, ATAC-seq, TFAP2A

ChIP-seq, and TT-seq reads mapped to STARR-seq peak overlapping MER11B elements in GP5d cells were extracted and mapped to the MER11B consensus sequence. Metaplots show the compiled signals at the consensus sequence. **b,** ATAC-seq, NFYA ChIP-seq and GRO-seq reads mapped to STARR-seq peak overlapping LTR12C elements in HepG2 cells were extracted and mapped to the LTR12C consensus sequence. Metaplots show the compiled signals at the consensus sequence. **c,** TFAP2A depletion has strong growth inhibitory effect on GP5d cells. DepMap CRISPR screening data for TFAP2A was plotted for colorectal and liver cell lines. Number of colorectal (n = 55) and liver (n = 20) cell lines are shown on top. X-axis shows the CRISPR Chronos score (TFAP2A gene effect), and Y-axis shows the TFAP2A expression. Source data are provided as a Source Data file.

Supplementary Figure 9

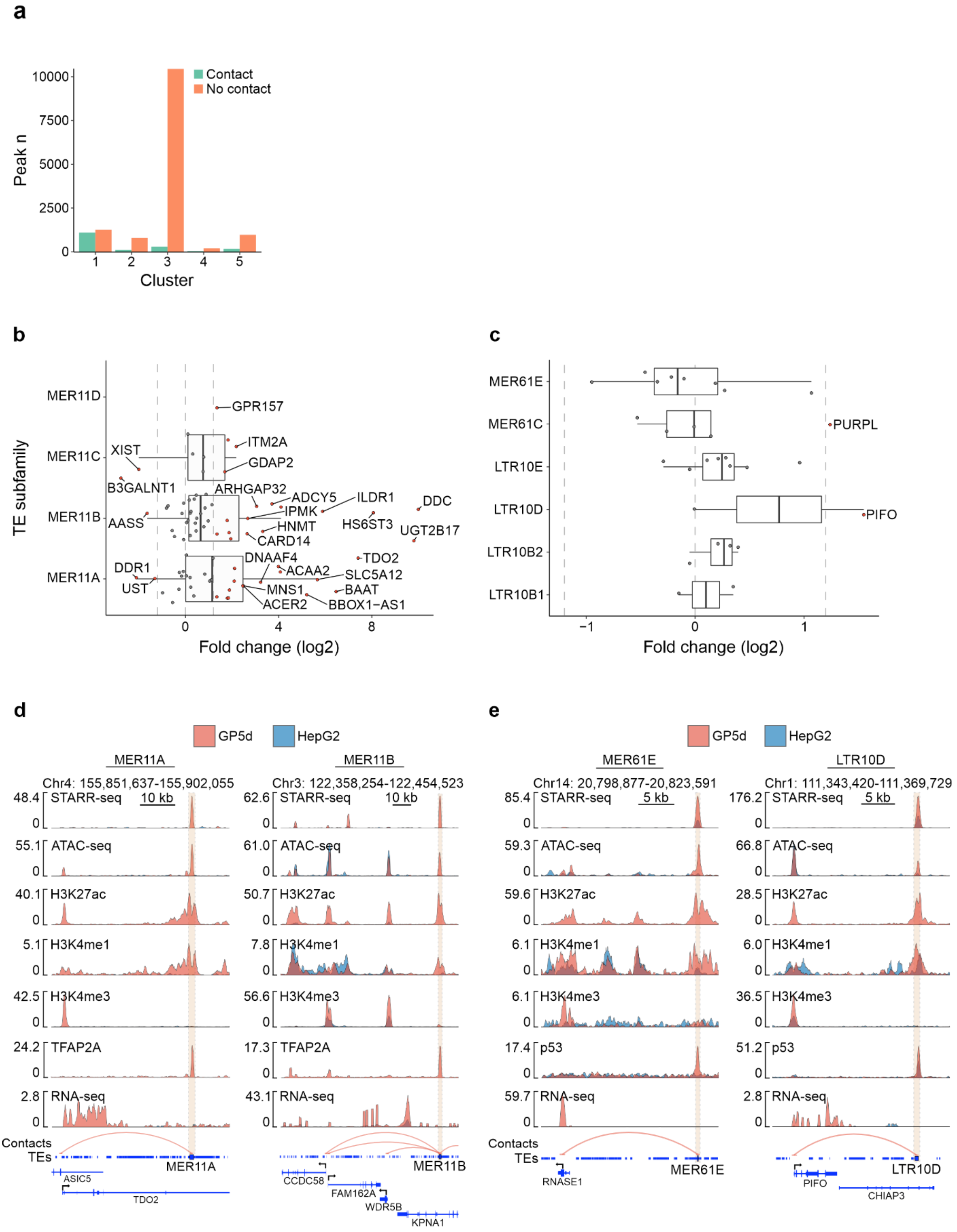

Supplementary Figure 9 | Predicting target genes of TE enhancers using activity-by contact (ABC) model. a, The number of STARR-seq peaks from different clusters in GP5d cells (from main **Fig. 2a**) with at least one predicted contact and without predicted contacts with gene promoters from the ABC model (see Methods). **b,** Differential expression of genes associated to MER11 subfamilies through ABC-predicted contacts, showing $\log_2$ fold change

from RNA-seq data from GP5d vs. normal colon epithelial (HCoEpiC) cells. The lower and upper hinges of the boxes represent the 25th to 75th percentiles, the midline is the median, and the whiskers extend from the hinges to the minimum and maximum values by 1.5 * IQR (interquartile range, i.e. distance between the 25th and 75th percentiles). (n = 32, 39, 9 and 1 for MER11A, MER11B, MER11C and MER11D, respectively) **c,** Differential expression of genes associated to p53-specifc subfamilies through ABC-predicted contacts, showing $\log_2$ fold change from RNA-seq data from GP5d wild type vs. GP5d p53-null cells. The boxplot features are as in panel **b.** (n = 2, 4, 2, 8, 5, and 8 for LTR10B1, LTR10B2, LTR10D, LTR10E, MER61C and MER61E, respectively) **d,** Genome browser views of active MER11A and MER11B elements, with tracks showing signals for STARR-seq, ATAC-seq, histone and TFAP2A ChIP-seq, RNA-seq and predicted gene targets from the ABC model. The signals at the loci are plotted for both GP5d and HepG2. To note, HepG2 lacks the active signals at the elements and the predicted gene targets show low expression in HepG2 despite the epigenetic marks of active promoters (ATAC, H3K4me3). **e,** Genome browser view of two overexpressed genes in GP5d WT vs. HepG2 cells (main **Fig. 6b**), *PIFO* and *RNASE1*, with predicted contacts to p53-specific MER61E and LTR10D elements, respectively. The lower STARR-seq and p53 ChIP-seq signals in HepG2 vs. GP5d cells correlate with lower expression of the genes. Source data are provided as a Source Data file.

Supplementary Figure 10

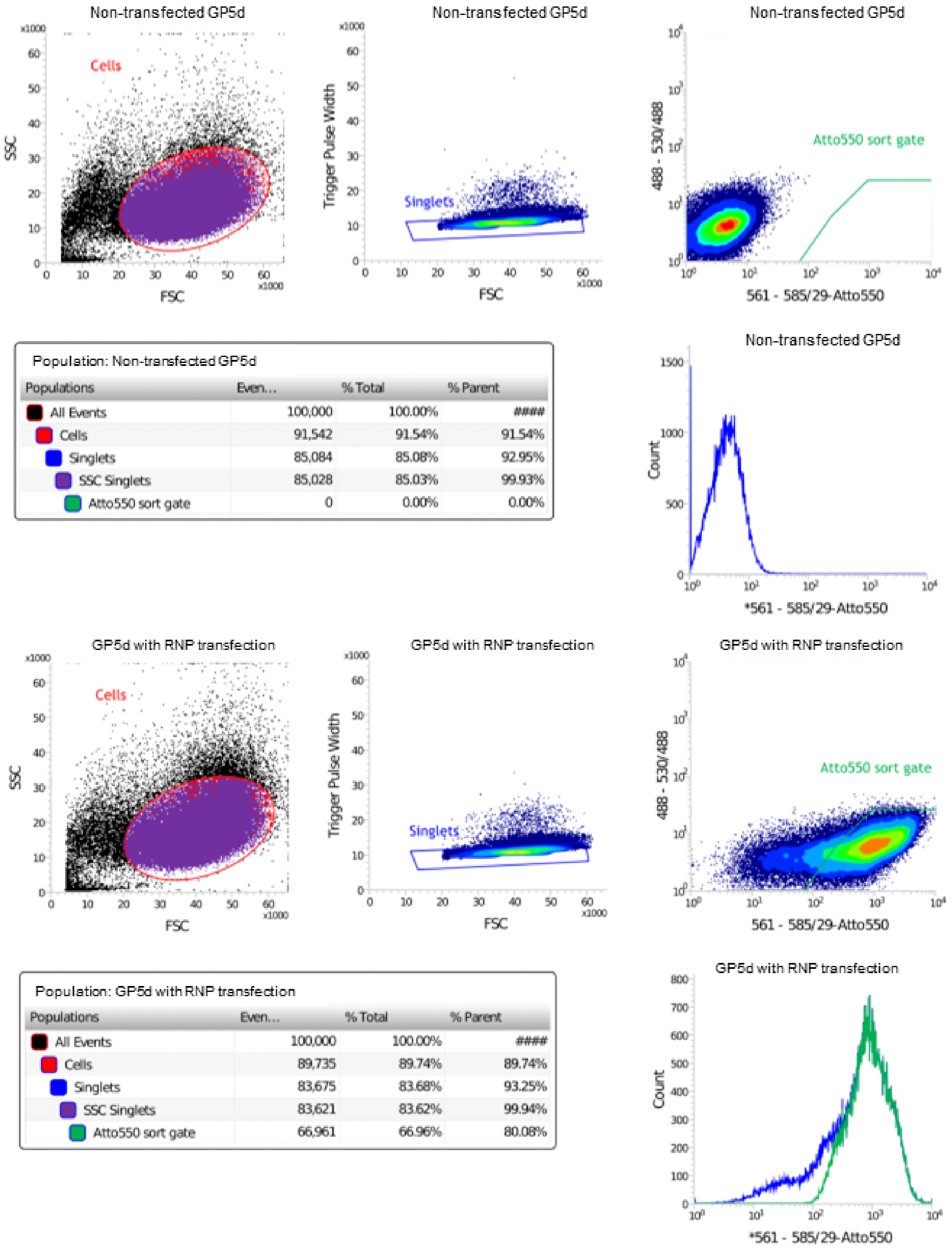

Population: Non-transfected GP5d

Populations	Even...	% Total	% Parent
All Events	100,000	100.00%	####
Cells	91,542	91.54%	91.54%
Singlets	85,084	85.08%	92.95%
SSC Singlets	85,028	85.03%	99.93%
Atto550 sort gate	0	0.00%	0.00%

Population: GP5d with RNP transfection

Populations	Even...	% Total	% Parent
All Events	100,000	100.00%	####
Cells	89,735	89.74%	89.74%
Singlets	83,675	83.68%	93.25%
SSC Singlets	83,621	83.62%	99.94%
Atto550 sort gate	66,961	66.96%	80.08%

Supplementary Figure 10 | Flow cytometry analysis for RNP-transfected GP5d cells. Flow cytometry analysis was used for measuring the RNP transfection efficiency of GP5d cells and for sorting single ATTO550-positive cells on 96-well plates for generating clonal cell lines for TE enhancer deletions, as tracrRNA used for generating the RNP complexes contain ATTO550 fluorochrome. Manual gating was performed using non-transfected GP5d cells (top panel), and similar gates were applied for RNP-transfected samples (lower panel for

represents RNP targeting MER11A overlapping STARR-seq peak (chr2:138425380-138427071). Gating strategy from left to right: 1. FSC/SSC: Cells were gated on the main population to exclude clear outliers such as cell debris. 2. FSC/Trigger pulse width: Cells were gated on the main population that represent single cells, excluding the outliers with larger trigger pulse width representing potential duplets. 3. Fluorescence was monitored on two channels: excitation 488 nm, emission 530/40 nm as an extra negative control, and excitation 561 nm, emission 585/29 nm for ATTO550. Gate was set using the non-transfected GP5d cells so that all cells remained negative for ATTO550. Same gate was maintained for analysis and sorting of RNP transfected cells.

Supplementary Figure 11

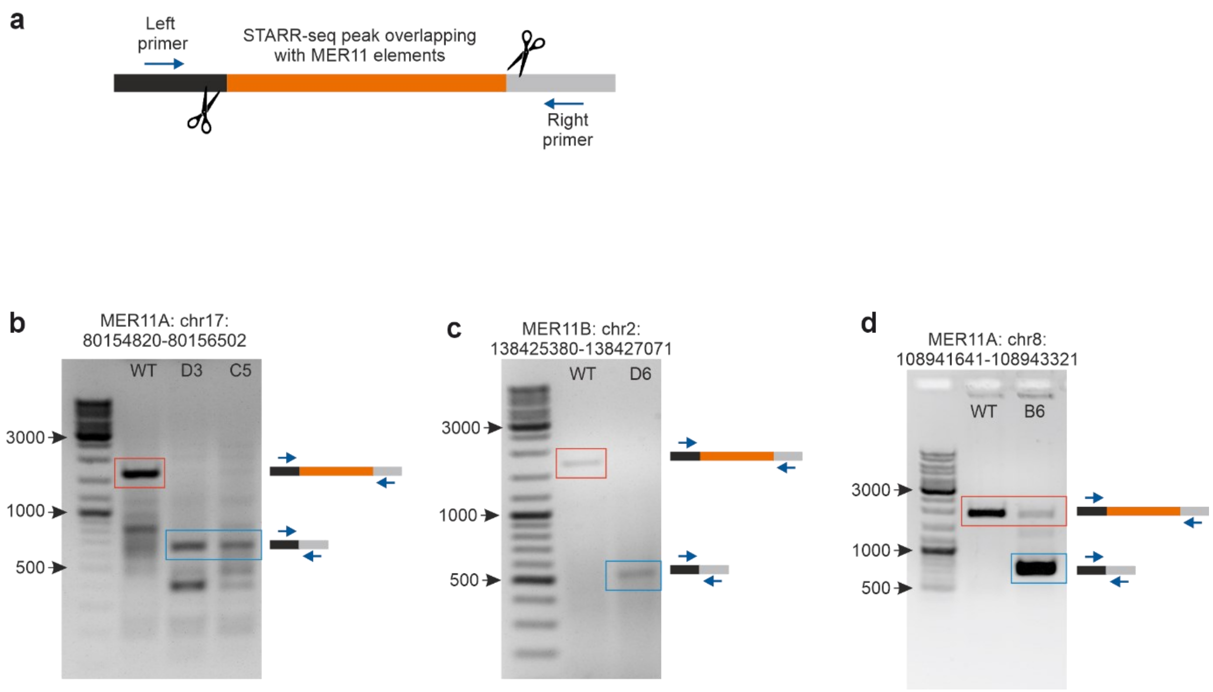

Supplementary Figure 11 | PCR validation of CRISPR/Cas9 mediated knock-out of MER11 elements. a, Schematic representation showing MER11-overlapping STARR-seq peaks, guide-RNA sites for Cas9 cutting sites (scissors), and the gDNA primers flanking the STARR-seq peaks (blue arrow) used for genotyping**. b,** Analysis of CRISPR deletion of MER11B element (chr17:80154820-80156502), using gel electrophoresis after genomic PCR. Expected wild-type (WT) amplicon size is 1982 bp. Expected knock-out (KO) amplicon size is ~714 bp. **c,** Analysis of CRISPR deletion of MER11B element (chr2:138425380-138427071), using gel electrophoresis after genomic PCR. Expected WT amplicon size is 1695 bp. Expected KO amplicon size is ~540 bp. **d,** Analysis of CRISPR deletion of MER11B element (chr8:108941641-108943321, heterozygous clone) using gel electrophoresis after genomic PCR. Expected WT amplicon size is 1920 bp. Expected KO amplicon size is ~748 bp. Uncropped gel images are provided in **Supplementary Figure 13**. Gel electrophoresis in Supplementary Figures 11b, 11c and 11d was performed twice to confirm genotype of selected clones.

Supplementary Figure 12

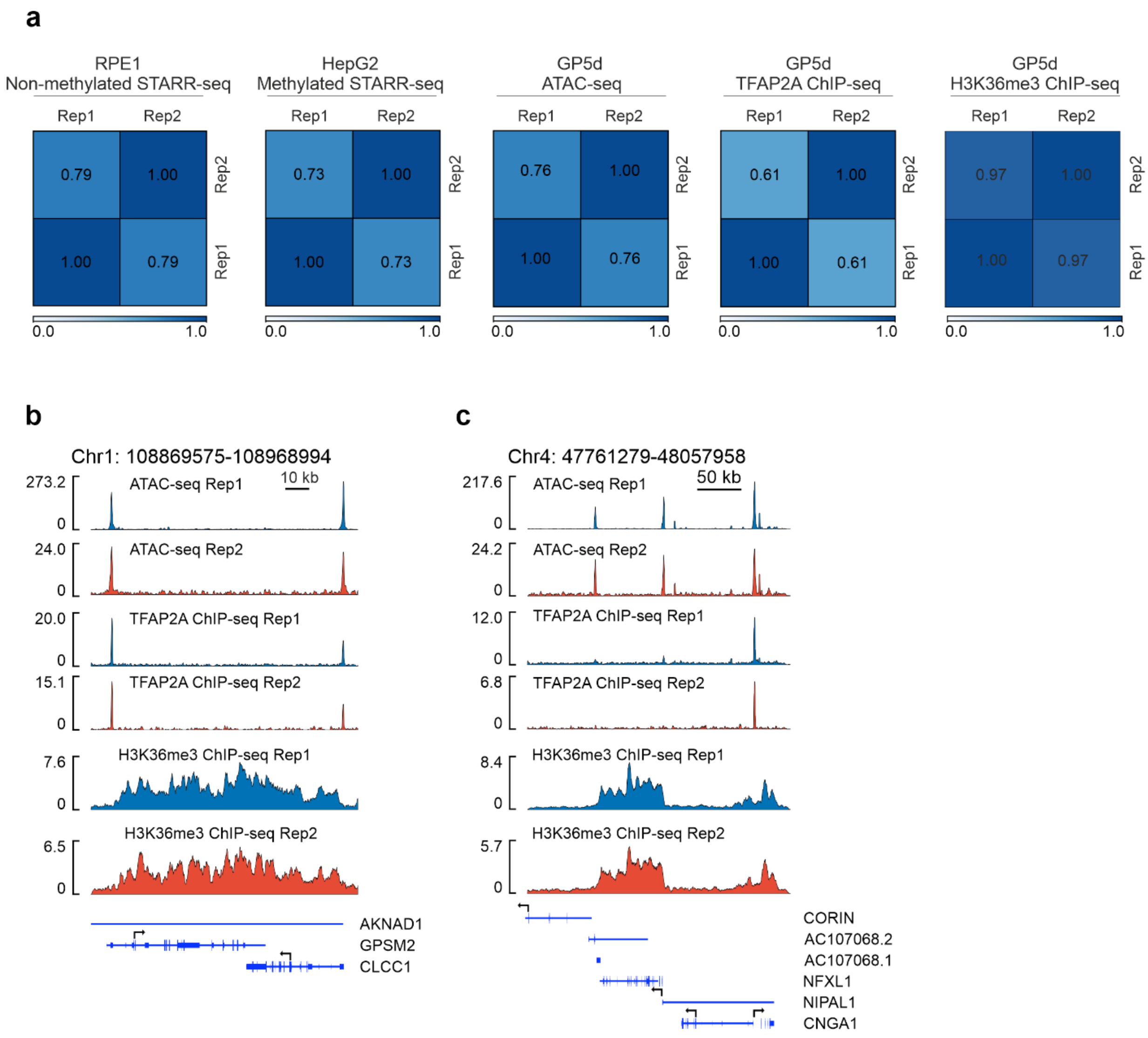

Supplementary Figure 12 | Quality control of STARR-seq, ATAC-seq and ChIP-seq samples. a, Correlation plots for replicates of RPE1 STARR-seq and methylated HepG2 STARR-seq, GP5d ATAC-seq and ChIP-seq for TFAP2A and H3K36me3. STARR-seq samples were technical replicates and ATAC-seq and ChIP-seq samples were biological replicates. Pearson's R is shown in the heatmap figures. **B-c,** Example browser snapshots of a genomic regions for the biological replicates of GP5d ATAC-seq and ChIP-seq for TFAP2A and H3K36me3.

Supplementary Figure 13

Supplementary Figure 11b

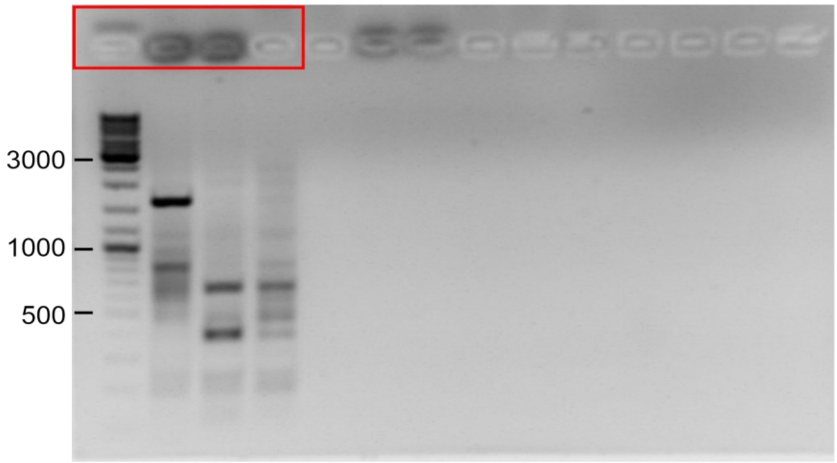

Supplementary Figure 11c

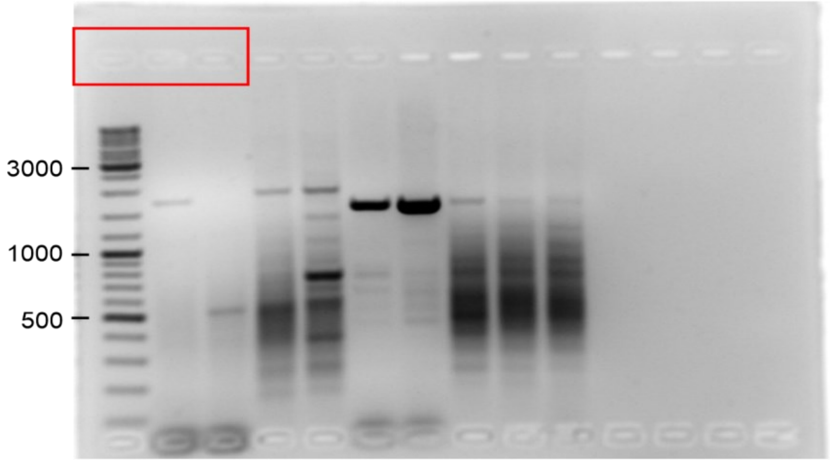

Supplementary Figure 11d

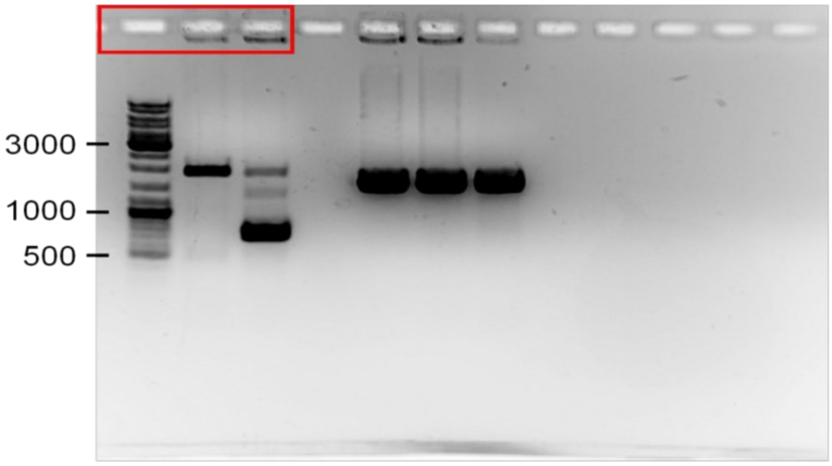

Supplementary Figure 13 | Source data for gel images. Uncropped gels images for Supplementary Figure 11b-d.

Supplementary References

1. Kapusta, A. et al. Transposable Elements Are Major Contributors to the Origin, Diversification, and Regulation of Vertebrate Long Noncoding RNAs. PLOS Genetics 9, e1003470 (2013).
2. Vierstra, J. et al. Global reference mapping of human transcription factor footprints. Nature 583, 729-736 (2020).
3. Wang, T. et al. Species-specific endogenous retroviruses shape the transcriptional network of the human tumor suppressor protein p53. Proceedings of the National Academy of Sciences of the United States of America 104, 18613-18618 (2007).
4. Kundaje, A. et al. Integrative analysis of 111 reference human epigenomes. Nature 518, 317-330 (2015).
5. Corces, M.R. et al. The chromatin accessibility landscape of primary human cancers. Science 362, eaav1898 (2018).
6. Hoadley, K.A. et al. Cell-of-Origin Patterns Dominate the Molecular Classification of 10,000 Tumors from 33 Types of Cancer. Cell 173, 291-304.e6 (2018).
7. Malta, T.M. et al. Machine Learning Identifies Stemness Features Associated with Oncogenic Dedifferentiation. Cell 173, 338-354.e15 (2018).
