# Supplementary material for: Transposable elements as tissue-specific enhancers in cancers of endodermal lineage
Source: Nat Commun. 2023 Sep 1;14:5313. doi: 10.1038/s41467-023-41081-4 (PMC10474299; doi:10.1038/s41467-023-41081-4)
Supplement: Supplementary file 3 — Description of Additional Supplementary Files [file 41467_2023_41081_MOESM3_ESM.pdf]

Description of Additional Supplementary Data provided as separate sheets in the Karttunen_Patel_et_al_Supplementary_Data_NCOMMS-22-52907B.xlsx document:

Supplementary Data 1: GP5d wild type non-methylated, methylated and GP5d non-methylated p53-KO STARR-seq peaks in MACS2 narrowpeak format, hg38 genome annotation, last column is the assigned cluster for each peak.

Supplementary Data 2: HepG2 non-methylated and methylated STARR-seq peaks in MACS2 narrowpeak format, hg38 genome annotation, last column is the assigned cluster for each peak.

Supplementary Data 3: RPE1 non-methylated STARR-seq peaks in MACS2 narrowpeak format, hg38 genome annotation, last column is the assigned cluster for each peak.

Supplementary Data 4: Results of the STARR-seq peak summit overlap analysis for TEs in GP5d, HepG2 and RPE1.

Supplementary Data 5: Full results of the TF motif analysis in all significant common and differentially enriched TE subfamilies in GP5d and HepG2 that overlapped a STARR-seq peak.

Supplementary Data 6: Predicted enhancer-gene contacts in GP5d from the ABC model. Subset of predicted contacts with overlapping STARR-seq peak summits and including overlapping TEs.

Supplementary Data 7: Read counts, mapping, and peak calling statistics for all STARR-seq datasets.

Supplementary Data 8: List of data used (generated in this study as well as publicly available) and annotations in the study, including publications and accessions for GEO/ENCODE.

Supplementary Data 9: List of guide-RNAs and primers used in this study.
