# Supplementary material for: Transposable elements as tissue-specific enhancers in cancers of endodermal lineage
Source: Nat Commun. 2023 Sep 1;14:5313. doi: 10.1038/s41467-023-41081-4 (PMC10474299; doi:10.1038/s41467-023-41081-4)
Supplement: Supplementary file 5 — Reporting Summary [file 41467_2023_41081_MOESM5_ESM.pdf]

Corresponding author(s): Biswajyoti Sahu

Last updated by author(s): Aug 15, 2023

Reporting Summary

Nature Portfolio wishes to improve the reproducibility of the work that we publish. This form provides structure for consistency and transparency in reporting. For further information on Nature Portfolio policies, see our Editorial Policies and the Editorial Policy Checklist.

Statistics

For all statistical analyses, confirm that the following items are present in the figure legend, table legend, main text, or Methods section.

n/a	Confirmed	
☐	☒	The exact sample size (*n*) for each experimental group/condition, given as a discrete number and unit of measurement
☐	☒	A statement on whether measurements were taken from distinct samples or whether the same sample was measured repeatedly
☐	☒	The statistical test(s) used AND whether they are one- or two-sided<br>*Only common tests should be described solely by name; describe more complex techniques in the Methods section.*
☒	☐	A description of all covariates tested
☐	☒	A description of any assumptions or corrections, such as tests of normality and adjustment for multiple comparisons
☐	☒	A full description of the statistical parameters including central tendency (e.g. means) or other basic estimates (e.g. regression coefficient) AND variation (e.g. standard deviation) or associated estimates of uncertainty (e.g. confidence intervals)
☐	☒	For null hypothesis testing, the test statistic (e.g. *F*, *t*, *r*) with confidence intervals, effect sizes, degrees of freedom and *P* value noted<br>*Give P values as exact values whenever suitable.*
☒	☐	For Bayesian analysis, information on the choice of priors and Markov chain Monte Carlo settings
☒	☐	For hierarchical and complex designs, identification of the appropriate level for tests and full reporting of outcomes
☐	☒	Estimates of effect sizes (e.g. Cohen's *d*, Pearson's *r*), indicating how they were calculated

Our web collection on statistics for biologists contains articles on many of the points above.

Software and code

Policy information about availability of computer code

Data collection	No specialized software was used for data acquisition.
Data analysis	FastQC v.0.11.9 was used for quality control and determination of read lengths.<br><br>Trimmomatic v.0.39 was used in trimming input data from STARR-seq to match the read length of reporter output.<br><br>In the STARR-, ChIP-, and ATAC-seq data, Bowtie2 v.2.4.1 was used for mapping, Picard v.2.23.4 was used for marking duplicates and insert size analysis, Samtools v.1.7 was used for filtering reads, MACS2 v2.2.7.1 was used for peak calling, bedtools v.2.29.2 was used to subtract ENCODE blacklisted regions and calculating coverage, bedGraphToBigWig v.377 was used to create a bigwig file and deepTools v.3.5.0 was used to create a RPKM-normalized coverage file. For ATAC-seq, reads mapped to the mitochondrial genome were removed with removeChrom.py script (https://github.com/jsh58/harvard/blob/master/removeChrom.py). Correlation analysis between replicates was performed by using multiBigwigSummary v.3.1.3 and plotted with plotCorrelation v.3.1.3.<br><br>The nanopore data was mapped with minimap2 2.16, quality control was performed with nanoplot 1.20.0 and Samtools 1.9, and nanopolish v.0.11.1 (cpggpc_new_train branch in GitHub) was used to call CpG and GpC methylation. Methylation tables were created with mtsv2bedGraph.py and parseMethylbed.py scripts from Lee et al. Nat. Methods (2020). The resulting methylation tables were converted to bedGraph and bigwig formats utilizing bedGraphToBigWig v.377. The CpG methylation frequency tables were smoothed with bsseq v.1.28.0 in R.<br><br>For RNA-seq, pseudoalignment and counting was performed with Salmon v.1.8.0. Reads were aligned with STAR v.2.5.3a by using the SQuIRE

pipeline v. 0.9.9.92. Read counts were calculated with featureCounts v.2.0.1. Diffential expression analysis was performed with DESeq2 v.1.32.0.

TEtranscripts v.2.2.1 was run on the SQuIRE alignment output. Output from DESeq2 v1.32.0 were used for TE subfamily expression analysis. Telescope v.1.0.3.1 analysis was performed on SQuIRE alignment output. Output from DESeq2 v1.32.0 were used in subsequent analysis. Nearby genes were associated with GREAT v.4.0.4.

Hi-ChIP data was processed with HiC-Pro v3.1.0. allValidPairs output from HiC-Pro was converted to a .hic file with the hicpro2juicebox.sh script from HiC-Pro, with juicer tools v.1.22. KR-normalized matrices were extracted from the .hic file with juicebox_dump.py and powerlaw fit was calculated with the compute_powerlaw_fit_from_hic.py script from ABC v0.2.2.

GRO-seq reads were trimmed to remove A-stretches and filter short and low-quality reads using the Trim Galore v.0.6.7. Reads were aligned using bowtie2 v.2.2.5 and strand specific bigwig files were created with Samtools v.1.9.

Genome arithmetic for called peaks and summits was performed with GenomicRanges R package v.1.44.0. STARR-seq peak summits were shuffled with bedtoolsr v.2.29.0-3, excluding masked regions in from the BSgenome.Hsapiens.UCSC.hg38.masked v.1.3.993. Statistical significance for TE subfamily enrichment was calculated with the rstatix package v.0.7.0.

Bwtools v.1.0 was used to plot compiled signal for ATAC-seq, ChIP-seq and TT-seq/GRO-seq at LTR consensus sequences.

All motif enrichments for each TE subfamily or STARR cluster were analyzed with AME from the MEME suite v. 5.0.2.

deepTools v.3.5.0 computeMatrix was used to compute a read matrix for the RPKM normalized bigwig files. The matrix was clustered with R kmeans function.

ABC v.0.2.2 was used for the prediction of enhancer-promoter contacts.

Statistical analysis was performed in R v.4.1.2. Profile plots were created from the bigwig files with the R package soGGi v.1.24.1. The signal was smoothed with Zoo package v.1.8-10. Genomic annotation for STARR-seq peaks was performed with ChIPseeker v.1.28.3. All plotting was performed with ggplot2 v.3.3.6 from the Tidyverse suite v.1.3.1 94. Motif enrichments were plotted with ComplexHeatmap v.2.8.0 95 and enrichment heatmaps that were plotted with EnrichedHeatmap v.1.22.0. GraphPad Prism v.9 (GraphPad) was used for Statistical analysis for Figure 4g, 6d and Extended data Figure 8c.

Custom scripts used in the analyses are provided in: https://github.com/Karttune/Karttunen_Patel_et_al.

For manuscripts utilizing custom algorithms or software that are central to the research but not yet described in published literature, software must be made available to editors and reviewers. We strongly encourage code deposition in a community repository (e.g. GitHub). See the Nature Portfolio guidelines for submitting code & software for further information.

Data

Policy information about availability of data

All manuscripts must include a data availability statement. This statement should provide the following information, where applicable:

- Accession codes, unique identifiers, or web links for publicly available datasets
- A description of any restrictions on data availability
- For clinical datasets or third party data, please ensure that the statement adheres to our policy

Data generated in this study has been deposited into GEO under accession GSE221053. The publicly available data was accessed as follows:

GP5d and HepG2 STARR-seq data was acquired from under GEO accession GSE180158. For GP5d, genomic and p53-null STARR-seq data were downloaded (GSM5454433 and GSM5454435) and for HepG2 genomic STARR replicates 1 and 2 were downloaded (GSM5454437 and GSM5454438).

GP5d ChIP-seq data for H3K27ac (GSM5454417), H3K9me3 (GSM5454420), H3K27me3 (GSM5454428), 5-FU treated mIgG (GSM5454414), untreated p53 (GSM5454412) and 5-FU treated p53 (GSM5454413) were acquired from the same study. GP5d H3K4me1 (GSM1240814) was obtained with the GEO accession GSE51234.

HepG2 ChIP-seq data for H3K27ac, H3K27me3, H3K36me3, H3K4me1, H3K4me3, H3K9me3, p53 and NFYA replicate 1 and 2 were downloaded from ENCODE (https://www.encodeproject.org/) with fastq file accessions ENCFF000BFD, ENCFF001FLQ, ENCFF001FMA, ENCFF000BEX, ENCFF901NZE, ENCFF000BFK, ENCFF257UIJ and ENCFF081VHA, respectively. Replicate 1 for HepG2 ATAC-seq was downloaded with accessions ENCFF664UPL and ENCFF289UIB for read file 1 and 2 respectively (https://www.encodeproject.org/).

GP5d TT-seq data was downloaded from GEO database under accession code GSM4610669. HepG2 GRO-seq raw data were downloaded from GEO database under accession code GSM2428726 (SRR5109940).

RPE1 ATAC-seq (GSM5366618) and ChIP-seq data for H3K27ac (GSM5345550), H3K27me3 (GSM5345534), H3K36me3 (GSM5345454), H3K4me1 (GSM5345374), H3K4me3 (GSM5345406), H3K9me3 (GSM5345502), were acquired from GSE175752 and p53 (GSM2677386) ChIP-seq was acquired from GSE100292. WGBS (GSM3394824) data for RPE1 was acquired from GSE120140.

Three replicates for HepG2 RNA-seq (ERR6351780, ERR6351781 and ERR6351782) were downloaded under ENA accession PRJEB3126260.

NCBI genome annotation files for GRCh38 were downloaded from Illumina iGenomes (http://igenomes.illumina.com.s3-website-us-east-1.amazonaws.com/Homo_sapiens/NCBI/GRCh38/Homo_sapiens_NCBI_GRCh38.tar.gz).

A gene annotation GTF file was acquired from Gencode Release 36 for the reference chromosomes (https://ftp.ebi.ac.uk/pub/databases/gencode/Gencode_human/release_36/gencode.v36.annotation.gtf.gz). The GTF file was transferred into a BED file with gtfToBed.sh (github.com/timplab/nanoNOMe/blob/isac/analysis/annotations/gtfToBed.sh) and a TSS and a gene body BED files were created with a script adapted from https://github.com/isaclee/nanoNOMe/blob/master/snakemake/downloaded_data_parse.smk.

A repeatMasker.txt (2021-09-03) file was downloaded from the UCSC table browser (https://genome.ucsc.edu/cgi-bin/hgTables). Only transposable element-derived repeat classes (LINE, SINE, LTR, and DNA) were retained and a file in BED format was created from the table, totaling 4745258 annotated repeats. MER11B and LTR12C consensus sequences were acquired from RepBase (https://www.girinst.org/repbase/update/index.html).

GRCh38 chromosome sizes file (2020-03-13) file was downloaded from UCSC (https://hgdownload-test.gi.ucsc.edu/goldenPath/hg38/bigZips/latest/). Unified GRCh38 blacklist BED file (ENCFF356LFX, release 2020-05-05) was downloaded from ENCODE project (https://www.encodeproject.org/).

Transcription factor motifs were acquired from JASPAR 2022 CORE non-redundant vertebrate annotations (https://jaspar.genereg.net/download/data/2022/CORE). The position weight matrices in MEME format were used for downstream motif analyses. Motif clustering data was downloaded from (https://resources.altius.org/~jvierstra/projects/motif-clustering-v2.0beta/).

Predicted LTR p53 binding site percentages were downloaded from Wang et al. PNAS (2007). TE age estimation data were downloaded from the TEanalysis pipeline, (https://github.com/4ureliek/TEanalysis/blob/master/Data/20141105_hg38_TEage_with-nonTE.txt).

TCGA cancer-type specific ATAC-seq peak sets were acquired from https://gdc.cancer.gov/about-data/publications/ATACseq-AWG.

Roadmap 25-state chromatin model bed files for 127 cell types were acquired from https://egg2.wustl.edu/roadmap/data/byFileType/chromhmmSegmentations/ChmmModels/imputed12marks/jointModel/final/.

Human research participants

Policy information about studies involving human research participants and Sex and Gender in Research.

Reporting on sex and gender	N/A
Population characteristics	N/A
Recruitment	N/A
Ethics oversight	N/A

Note that full information on the approval of the study protocol must also be provided in the manuscript.

Field-specific reporting

Please select the one below that is the best fit for your research. If you are not sure, read the appropriate sections before making your selection.

☒ Life sciences ☐ Behavioural & social sciences ☐ Ecological, evolutionary & environmental sciences

For a reference copy of the document with all sections, see nature.com/documents/nr-reporting-summary-flat.pdf

Life sciences study design

All studies must disclose on these points even when the disclosure is negative.

Sample size	The complexity estimates and quality controls of the STARR-seq libraries are described in Sahu et al. Nat. Gen. (2022). The biological conclusions made from the achievable sample size was ensured by i) manual inspection of the genomic peaks ii) statistical testing, and iii) replicate comparison. The final read counts and called peaks for the STARR-seq libraries after quality control, removing duplicates and mapping quality filtering are listed in the supplementary tables.
Data exclusions	ENCODE blacklisted regions were removed from all the peak files used in the analysis. Low-quality reads were filtered out from all used sequencing data.<br>In the repeatMasker annotation, only major classes of transposable elements (LINE, LTR, SINE, DNA) were retained for further analysis.
Replication	HepG2 STARR-seq (non-methylated) was performed in two technical replicates and GP5d STARR-seq was performed in 4 different conditions (WT and p53-null, non-methylated and methylated) as described in Sahu et al. Nat. Gen. (2022), of which 2 conditions were used in this study. HepG2 STARR-seq (methylated), RPE1 STARR-seq (non-methylated), GP5d TFAP2A ChIP-seq and GP5d H3K36me3 ChIP-seq were performed in two replicates. RNA-seq data was performed in triplicates. For GP5d ATAC-seq, replicate 1 was from this study and replicate 2 was from Sahu et al. Nat. Gen. (2022). All attempts for replication were successful, and Pearson correlation analysis for replicate experiments shown in Extended Data Fig. 12.
Randomization	As experiments were performed on uniform biological material such commercial human cell lines, randomization of experimental groups was

	not applicable. For statistical analysis of TE enrichment at STARR-seq peak summits, the peak summits were randomly shuffled 1000 times in the analysis to estimate a random distribution of peak summits.
Blinding	Analysis was performed using large sequencing datasets representing whole human genome, thus blinding of the investigators was not relevant to this study.

Reporting for specific materials, systems and methods

We require information from authors about some types of materials, experimental systems and methods used in many studies. Here, indicate whether each material, system or method listed is relevant to your study. If you are not sure if a list item applies to your research, read the appropriate section before selecting a response.

Materials & experimental systems

n/a	Involved in the study
☐	☒ Antibodies
☐	☒ Eukaryotic cell lines
☒	☐ Palaeontology and archaeology
☒	☐ Animals and other organisms
☒	☐ Clinical data
☒	☐ Dual use research of concern

Methods

n/a	Involved in the study
☐	☒ ChIP-seq
☐	☒ Flow cytometry
☒	☐ MRI-based neuroimaging

Antibodies

Antibodies used	H3K4me3 (Sigma-Aldrich, 07-473), H3K36me3 (Diagenode, C1541092), H3K27ac (Diagenode, C15410196), TFAP2A (abcam, AB52222), mouse IgG (Santa Cruz Biotechnology, sc-2027) and rabbit IgG (Santa Cruz Biotechnology, sc-2025). ChIP-seq was performed by using 2 µg of antibody per reaction.
Validation	The anti-H3K4me3 polyclonal antibody is raised in rabbit against the region of histone H3 containing trimethylated lysine 4. It is recommended for detecting H3K4me3 in ChIP-experiments in human by the manufacturer with >900 citations available for this antibody (https://www.sigmaaldrich.com/FI/en/product/mm/07473).<br><br>The anti-H3K36me3 polyclonal antibody is raised in rabbit against the region of histone H3 containing trimethylated lysine 36. It is recommended for detecting H3K36me3 in ChIP-experiments in human by the manufacturer, and there are validation data and >20 citations available for this antibody (https://www.diagenode.com/en/p/h3k36me3-polyclonal-antibody-premium-sample-size-10-ug).<br><br>The anti-H3K27ac polyclonal antibody is raised in rabbit against the region of histone H3 containing aceylation at lysine 27. It is recommended for detecting H3K27ac in ChIP-experiments in human by the manufacturer, and there is validation data and >80 citations available for this antibody (https://www.diagenode.com/en/p/h3k27ac-polyclonal-antibody-premium-50-mg-18-ml).<br><br>The anti-Transcription factor AP-2-alpha (TFAP2A) polyclonal antibody is raised in rabbit against a synthetic peptide corresponding to Human TFAP2A. It is recommended for detecting human TFAP2A by the manufacturer and >20 citations are available for this antibody (https://www.abcam.com/transcription-factor-ap-2-alpha-antibody-ab52222.html).<br><br>The mouse IgG antibody is an affinity purified isotype control immunoglobulin from mouse. It is recommended for use as an isotype control immunoglobulin for ChIP-seq, and >1000 citations available for this antibody (https://www.scbt.com/p/normal-mouse-igg)<br><br>The rabbit IgG antibody is an affinity purified isotype control immunoglobulin from rabbit. It is recommended for use as an isotype control immunoglobulin for ChIP-seq, and >16 citations available for this antibody (https://datasheets.scbt.com/sc-2027.pdf)

Eukaryotic cell lines

Policy information about cell lines and Sex and Gender in Research

Cell line source(s)	Colon cancer cell line GP5d (Sigma, 95090715), HepG2 (ATCC, HB-8065), HCoEpiC (ScienCell, 2950) and RPE1 (ATCC, CRL-4000)
Authentication	All cell lines were directly obtained from trusted vendors (Sigma, ATCC and SciencCell) and low-passage cells were used in experiments. Vendors such as ATCC perform authentication and quality-control tests on all distribution lots, so the cell lines were not re-authenticated by the user.
Mycoplasma contamination	All cell lines tested negative for mycoplasma contamination upon purchase and were routinely checked as per standard good laboratory practice.
Commonly misidentified lines (See ICLAC register)	Cell lines used in this study are not in the list of commonly misidentified cell lines.

ChIP-seq

Data deposition

- [x] Confirm that both raw and final processed data have been deposited in a public database such as GEO.
- [x] Confirm that you have deposited or provided access to graph files (e.g. BED files) for the called peaks.

Data access links<br>*May remain private before publication.*	Raw and processed files have been deposited into GEO under accession GSE221053.
Files in database submission	GP5d_H3K36me3_S19_L002_R1_001.fastq.gz<br>GP5d_H3K4me3_S20_L002_R1_001.fastq.gz<br>GP5d_TFAP2A_S9_R1_001.fastq.gz<br>GP5D-mIgG_S10.fastq.gz<br>GP5d-rIgG_S2.fastq.gz<br>GP5d_H3K36me3_rep2_R1.fastq.gz<br>GP5d_TFAP2A_rep2_S87_R1_001.fastq.gz<br>GP5D_H3K36me3_peaks.broadPeak<br>GP5D_H3K36me3_rep2_peaks.broadPeak<br>GP5D_H3K4me3_peaks.broadPeak<br>GP5D_TFAP2A_peaks.narrowPeak<br>GP5D_TFAP2A_rep2_peaks.narrowPeak<br>GP5D_H3K36me3_final.bw<br>GP5d_H3K36me3_rep2_final.bw<br>GP5D_H3K4me3_final.bw<br>GP5D_TFAP2A_final.bw<br>GP5D_TFAP2A_rep2_final.bw<br>GP5D_mIgG_final.bw<br>GP5D_rIgG_final.bw
Genome browser session<br>(e.g. UCSC)	BigWig track files and peak files are deposited in GEO for loading into a genome browser.

Methodology

Replicates	One replicate was used for the previously unpublished ChIP-seq data used in this study.
Sequencing depth	GP5d H3K4me3, 20422556 final mapped reads<br>GP5d H3K36me3, replicate 1, 29197468 final mapped reads<br>GP5d H3K36me3, replicate 2, 25967183 final mapped reads<br>GP5d mIgG, 34898226 final mapped reads<br>GP5d, TFAP2A, replicate 1, 23785706 final mapped reads<br>GP5d, TFAP2A, replicate 2, 17989037 final mapped reads<br>GP5d, rIgG, 26371829 final mapped reads
Antibodies	H3K4me3 (Sigma-Aldrich, 07-473), H3K36me3 (Diagenode, C1541092-10), TFAP2A (abcam, AB52222), mouse IgG (Santa Cruz Biotechnology, sc-2027) and rabbit IgG (Santa Cruz Biotechnology, sc-2025). ChIP-seq was performed by using 2 µg of antibody per reaction.
Peak calling parameters	Peaks were called using MACS2 with options -f BAMPE -g hs --keep-dup all (broad peaks were called for H3K4me3 and H3K36me3, narrow peaks for TFAP2A)
Data quality	Fastqc v.0.11.9 was used for quality control of raw data, alignment statistics were checked, fraction of reads in peaks (FRiP) was calculated and data was manually inspected in a genome browser.
Software	Bowtie2 v.2.4.1 (Langmead and Salzberg, Nat Methods 9, 357-359, 2012)<br>Samtools 1.7 (Li et al., Bioinformatics, 25(16): 2078-2079, 2009)<br>Picard Tools v.2.23.4 (http://broadinstitute.github.io/picard/)<br>MACS2 v.2.2.7.1 (Zhang et al. Genome Biol. 9, pp. R137, 2008)

Flow Cytometry

Plots

Confirm that:

- [x] The axis labels state the marker and fluorochrome used (e.g. CD4-FITC).
- [x] The axis scales are clearly visible. Include numbers along axes only for bottom left plot of group (a 'group' is an analysis of identical markers).
- [x] All plots are contour plots with outliers or pseudocolor plots.
- [x] A numerical value for number of cells or percentage (with statistics) is provided.

Methodology

Sample preparation	GP5d cells were transfected with ribonucleoprotein (RNP) complex. Equimolar ratios of target-specific crRNAs and ATTO550-tracrRNA (IDT, 1075928) were annealed. RNP complex were constituted from Alt-R S.p. HiFi Cas9 Nuclease V3 (IDT, 1081060; 1,000ng per 200,000 cells) and target-specific sgRNA (250ng per 200,000 cells) and transfected to cells by using CRISPRMAX (Life Technologies, CMAX000003) according to manufacturer's protocol. GP5d cells were trypsinized 24 hours after transfection, washed once and resuspended in cold PBS. The flow cytometry analysis at the HiLife Flow Cytometry Unit, University of Helsinki, Finland, using BD Influx System (USB) and BD FACS software (version 1.2.0.142).
Instrument	BD Influx System (USB), model number X646500S7001
Software	BD FACS™ Software, 1.2.0.142
Cell population abundance	Out of 75,365 RNP transfected GP5d cells analyzed, 92.29% were singlets based on SSC/FSC, out of which 99.92% were ssc-singlets excluding the outliers with larger trigger pulse width representing potential duplets. Gate for ATTO550 was set so that all non-transfected cells were negative. Out of 69,500 singlets cells analyzed from the transfected sample, 80.16% were positive for ATTO550.
Gating strategy	Manual gating was performed using non-transfected GP5d cells, and similar gates were applied for RNP transfected samples to analyze transfection efficiency. Gating strategy is described in Extended Data Fig. 10 (top panels). Gating strategy from left to right: 1. FSC/SSC: Cells were gated on the main population to exclude clear outliers such as cell debris. 2. FSC/Trigger pulse width: Cells were gated on the main population that represent single cells, excluding the outliers with larger trigger pulse width representing potential duplets. 3. Fluorescence was monitored on two channels: excitation 488 nm, emission 530/40 nm as an extra negative control, and excitation 561 nm, emission 585/29 nm for ATTO550. Gate was set using the non-transfected GP5d cells so that all cells remained negative for ATTO550. Same gate was maintained to analyze RNP transfected cells to measure the proportion of ATTO550-positive cells.

- [x] Tick this box to confirm that a figure exemplifying the gating strategy is provided in the Supplementary Information.
