# Supplementary material for: Transposable elements as tissue-specific enhancers in cancers of endodermal lineage
Source: Nat Commun. 2023 Sep 1;14:5313. doi: 10.1038/s41467-023-41081-4 (PMC10474299; doi:10.1038/s41467-023-41081-4)
Supplement: Supplementary file 6 — Source Data [file 41467_2023_41081_MOESM6_ESM.zip › Source Data/Inventory of Source data files.docx]

**Description of source data files:**

- An excel file for main and Supplementary Figures (source_data.xlsx)
- Source data files for Figure 2a (fig_2a_source_data.tsv), Supplementary Figures 3a (left panel) (Supplementary_Figures_3a_left_panel_source_data.tsv) and Supplementary Figures 3a (right panel) (Supplementary_Figures_3a_right_panel_source_data) are large dataset, which are shared as separate text documents.
